# Psoas muscle size, possible sarcopenia and frailty, and long-term survival in elderly patients after isolated surgical aortic valve replacement for aortic stenosis

**DOI:** 10.1007/s12055-021-01253-7

**Published:** 2022-02-02

**Authors:** Yasuo Kondo, Tomoaki Suzuki, Masahide Enomoto, Noriyuki Takashima, Takeshi Kinoshita

**Affiliations:** Division of Cardiovascular Surgery, Shiga University of Medical Science, Setatsukinowa, Otsu, 520-2192 Japan

**Keywords:** Psoas muscles, Aortic valve stenosis, Isolated surgical aortic valve replacement, Elderly, Sarcopenia

## Abstract

**Purpose:**

This study investigated the use of psoas muscle area index (PAI) as an indicator of mortality risk in relation to survival in elderly patients after isolated surgical aortic valve replacement (SAVR) for aortic valve stenosis (AS).

**Methods:**

Between January 2005 and March 2015, 140 patients with AS, aged ≥ 70 years, and with preoperative abdominal computed tomography scans, underwent elective, primary, isolated SAVR. PAI showed the ratio of the psoas muscle cross-sectional area at the fourth lumbar vertebral level to body surface area, and PAI less than the gender-specific lowest 20th percentile we called “low PAI” for the purposes of this study. Patients were classified as low PAI (*n* = 29) or normal PAI (*n* = 111).

**Results:**

The mean age in the low-PAI group was significantly older than in the normal-PAI group (81.0 vs. 77.3 years; *p* = 0.001). The mean follow-up was 4.25 years. The low-PAI group had a lower survival rate than the normal-PAI group at 1 year (89.7 ± 5.7% vs. 96.3 ± 1.8%), at 3 years (71.6 ± 9.3% vs. 91.5 ± 2.7%), and overall (53.0 ± 13.4% vs. 76.0 ± 5.6%; *p* = 0.039). The prognostic factors of mortality included low PAI (hazard ratio 2.95; 95% confidence interval 1.084–8.079; *p* = 0.034).

**Conclusions:**

PAI was associated with reduced overall survival after isolated SAVR in elderly people. PAI measurement may help to predict patient risks.

## Introduction

Elderly patients are increasingly presenting for cardiac surgery worldwide. Older patients usually have several co-morbidities and additional factors resulting from age, generally called frailty, one aspect of which is sarcopenia (etymologically flesh/muscle poverty). Frailty has been seen as a general metric of health [1]. Frailty can be evaluated in various ways including questionnaires and measurements of hand grip strength and gait speed [2, 3]. Such measurements can vary in days or weeks [4], and frailty assessment tools are not standardized. Core muscle size can be easily and objectively assessed on computed tomography (CT) of the abdomen. Recently, several studies have shown that possible sarcopenia characterized by low muscle size, assessed by CT, may be associated with poor prognosis in patients undergoing abdominal aortic aneurysm repair [5], surgical and transcatheter aortic valve replacement (TAVR) [6, 7], and elective general and vascular surgery [8].

Assessment of core muscle size via CT measurement of psoas muscle volume or cross-sectional area has been shown to have significant prognostic value for patients undergoing TAVR, valve surgery, total arch replacement, and left ventricular assist device implantation [6, 9–13]. The psoas muscle area index (PAI) has also been found associated with postoperative mortality after surgical aortic valve replacement (SAVR) for aortic valve stenosis (AS), but those studies included concomitant surgery such as coronary artery bypass grafting [6, 7]. In patients undergoing SAVR, all-cause mortality tended to be worse in patients with concomitant coronary artery bypass grafting [14]. The primary aim was to examine the association of PAI with early and mid-term all-cause mortality in elderly patients after elective primary, isolated SAVR for AS. The secondary aim was to identify other predictors of all-cause mortality.

## Patients and methods

### Patient population

**Fig. 1:**
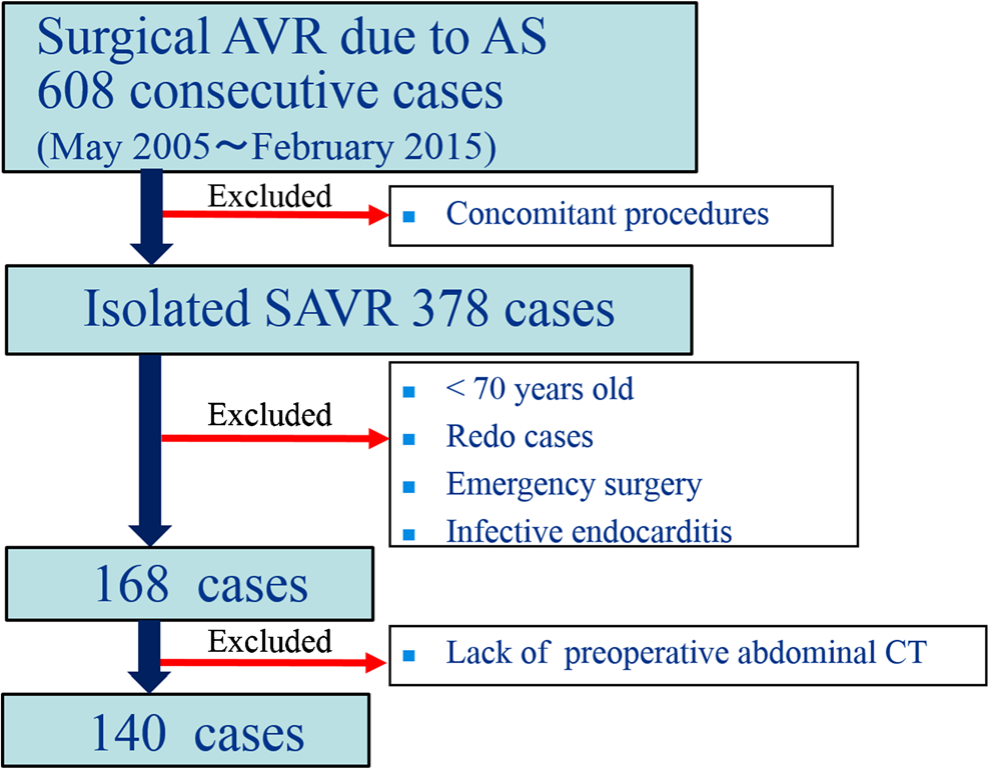
Patient selection diagram

Six hundred and eight consecutive patients underwent SAVR for AS between January 2005 and March 2015 in our institute. Excluding criteria were age under 70 years, lack of adequate preoperative CT imaging of the fourth lumbar (L4) vertebral level, emergency cases, redo cases, infective endocarditis cases, muscular and neurogenic disorder cases, or any concomitant procedure. In this study, 28 patients were excluded for lack of appropriate CT images. Since 2008, we have done abdominal CT for all potential SAVR/TAVR cases. After exclusions, a final cohort of 140 patients (mean age, 78.1 years) were included in this retrospective study (Fig. 1). TAVR has been available as an alternative to SAVR in our institution only since March 2016, so this study was not affected by any selection bias involving TAVR.

Study patients were followed in the outpatient clinic or by telephone contact. The follow-up rate was 90.7%. The last follow-up date was December 2017. We compared the early and long-term all-cause mortality of the two groups. The ethics committee of the Shiga University of Medical Science approved this retrospective study and waived patient consent on condition that no patients were identified. (Permit number: R2017-146).

### Surgical procedure

Standard median sternotomy and cardiopulmonary bypass established with the ascending aorta and the superior and inferior vena cava were used in all cases. The superior and inferior vena cava were snared to isolate the right atrium. A left ventricular vent through the right upper pulmonary vein was used. A retrograde cannula was inserted into the coronary sinus via right atriotomy and snared with a purse string suture placed shallow in the coronary sinus orifice, avoiding over-insertion of the cannula. After aortic cross-clamping, intermittent cold blood cardioplegia was infused through the retrograde cannula into the coronary sinus, and retrograde cardioplegia doses were delivered every 20–30 min through the coronary sinus. Core temperature was maintained at 32 °C by cardiopulmonary bypass. After excision of the aortic valve, the valve prosthesis was implanted in the supra-annular position. A bioprosthetic valve was used in all cases. All operations were performed or supervised by two expert surgeons.

### Measurement of psoas muscle area and definition of low PAI

**Fig. 2:**
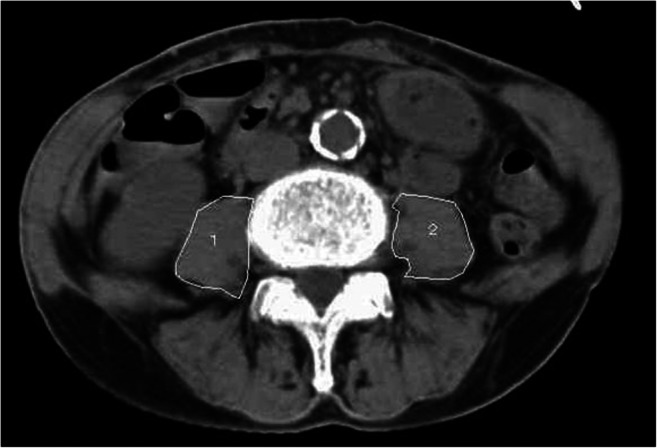
Measurement of the psoas muscle area on preoperative CT. Cross-sectional areas (mm^2^) of the psoas muscle at the level of the inferior border of the fourth lumbar vertebra (L4) measured by manual tracing on CT scans

Psoas muscle areas were measured on a single transaxial CT image by 2 experienced observers (cardiothoracic surgeons who were blinded to patient outcomes), and PAI was calculated as the average of their measurements. The paired *t* test and Pearson correlation coefficient both indicated low inter-observer variability between 2 observers (*r* ≥ 0.99, *p* < 0.001 excellent). Data were also analyzed using the inter-class coefficient (ICC) (2, 1) to determine inter-observer reliability of psoas size measurements. The ICC (2, 1) was 0.994 (C.I. 0.900–0.998 excellent). ShadeQuest/ViewR software (version 1.22.608, Yokogawa Medical Solutions Corporation, Tokyo, Japan) was used to manually trace the borders of the right and left psoas on axial images at the level of the inferior border of the L4 vertebra (Fig. 2). The total cross-sectional area of the psoas muscles was calculated as the sum of the areas of the resulting enclosed regions. Vasculature, fat, or other tissues within or around the psoas muscle area were included in the manually traced measurements. Body surface area (BSA) (m^2^) was determined by the Du Bois method: BSA = 0.007184 × height^0.725^ × weight^0.425^. PAI was determined by the equation: PAI = the total bilateral psoas muscle area at the level of the inferior border of the L4 (mm^2^) divided by the BSA (m^2^). In this study, the patients were classified as low PAI if they fell into the gender-specific lowest 20th percentile of PAI.

### Statistical analysis

Comparison of clinical characteristics between the two groups was performed using Pearson’s *χ*^2^ test for categorical variables, unpaired *t* test for normally distributed variables, and Mann–Whitney*U* test for skewed variables. To evaluate the effects of preoperative variables on postoperative mortality, preoperative variables with *p* values < 0.2 in univariate regression analysis were included in a multivariate Cox proportional hazards model. The estimated survival rates were calculated using the Kaplan–Meier method and compared using the log-rank test. All statistical testing was two-sided. Results were considered statistically significant at *p* < 0.05. All analyses were performed with SPSS version 20.0 (SPSS, Inc., Chicago, IL, USA) statistical software.

## Results

**Fig. 3:**
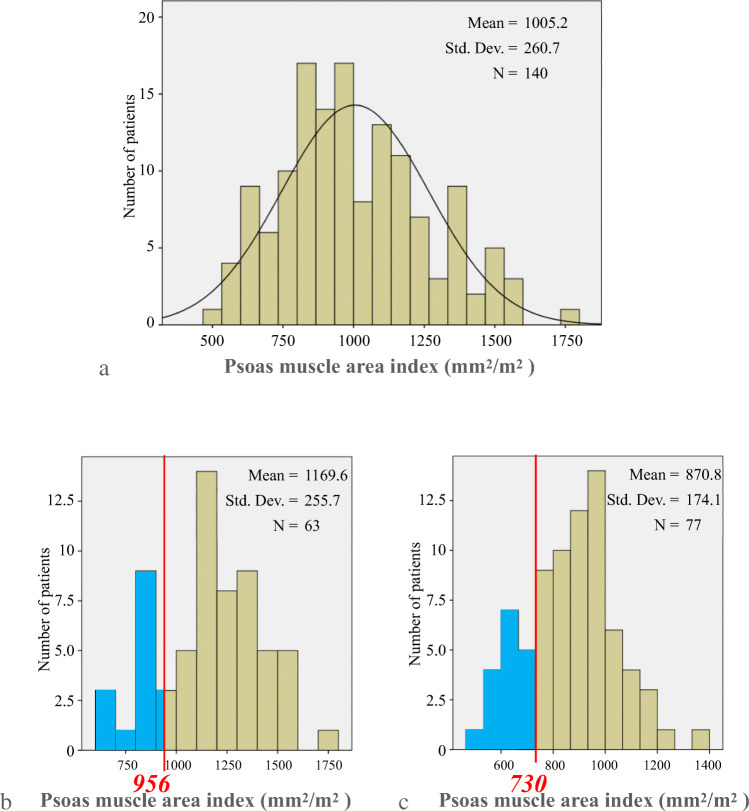
**a** Histogram of the PAI (all patients). **b** Histogram of the male PAI. **c** Histogram of the female PAI. The vertical red lines show the cut-off values for the gender-specific lowest 20th percentile of PAI. Males, 956 mm^2^/m^2^. Females, 730 mm^2^/m^2^

A histogram of PAI showed normal distribution (Fig. 3a). The average PAI values were 1169.6 ± 255.7 for males and 870.8 ± 174.1 mm^2^/m^2^ for females (Fig. 3b, c).

The cut-off value for the lowest 20th percentile PAI was 956 mm^2^/m^2^ for males, so that PAI ≤ 956 mm^2^/m^2^ was classified as low PAI, and PAI > 956 mm^2^/m^2^ meant normal PAI. The cut-off for females was 730 mm^2^/m^2^, so that PAI ≤ 730 mm^2^/m^2^ meant low PAI, and PAI > 730 mm^2^/m^2^ meant normal PAI. Thus, 140 patients were divided into 2 groups: the normal-PAI group (*n* = 111) and the low-PAI group (*n* = 29).

Patient characteristics are shown in Table 1. Compared with the normal-PAI group, the low-PAI group were significantly older (*p* = 0.001) and had smaller psoas muscle area and PAI (*p* < 0.001). Although no statistically significant difference was found, BSAs were slightly smaller in the low-PAI group.

**Table 1:** Preoperative characteristics by PAI status

Characteristics	Overall (*n* = 140)	Low PAI (*n* = 29)	Normal PAI (*n* = 111)	*p value*
Age at operation, years	78.1 ± 5.2	81.0 ± 5.8	77.3 ± 4.7	0.001
BSA, m^2^	1.49 ± 0.17	1.45 ± 0.15	1.50 ± 0.18	0.051
Body mass index, kg/m^2^	22.5 ± 3.8	21.6 ± 4.2	22.8 ± 3.7	0.156
Ejection fraction, %	61.9 ± 9.0	62.2 ± 9.0	62.1 ± 9.0	0.622
Aortic value area, cm^2^	0.80 ± 0.17	0.79 ± 0.15	0.80 ± 0.18	0.731
Mean transaortic pressure gradient, mmHg	51.0 ± 18.1	52.0 ± 19.4	50.8 ± 17.9	0.780
Serum albumin, mg/dl	3.9 ± 0.4	3.9 ± 0.3	3.9 ± 0.5	0.794
Serum creatinine, mg/dl	1.44 ± 1.9	1.34 ± 1.8	1.47 ± 1.9	0.761
EuroSCORE II, %	2.9 ± 1.9	3.3 ± 2.3	2.9 ± 1.9	0.316
Psoas muscle area, mm^2^	1520 ± 513	1025 ± 221	1648 ± 488	< 0.001
Psoas muscle area index, mm^2^/m^2^	1005 ± 260	704 ± 112	1083 ± 290	< 0.001
Gender				0.983
Female	77 (55.0)	16 (55.2)	61 (55.0)	
Male	63 (45.0)	13 (44.8)	50 (45.0)	
New York Heart Association				0.644
I	13 (9.3)	1 (3.6)	12 (10.9)	
II	89 (63.6)	18 (64.3)	69 (62.7)	
III	28 (20.0)	7 (25.0)	21 (19.1)	
IV	10 (7.1)	2 (7.1)	8 (7.3)	
Comorbid disease				
Diabetes	32 (22.9)	7 (24.1)	25 (22.7)	0.872
Dyslipidemia	51 (36.4)	10 (34.5)	41 (36.9)	0.807
Hypertension	90 (64.3)	15 (51.7)	75 (67.6)	0.113
CKD (cre > 1.5 mg/dl)	19 (13.6)	3 (10.3)	16 (14.4)	0.569
End-stage renal disease	11 (7.9)	2 (6.9)	9 (8.1)	0.829
Peripheral vascular disease	6 (4.3)	2 (6.9)	4 (3.6)	0.436
Previous cerebrovascular accidents	5 (3.6)	1 (3.4)	4 (3.6)	0.968
Previous percutaneous coronary intervention	15 (10.8)	3 (10.3)	12 (10.8)	0.942
Arrhythmia	8 (5.7)	2 (6.9)	6 (5.4)	0.758
COPD (FEV 1.0 < 70%)	44 (31.4)	11 (37.9)	33 (30.0)	0.414
Current smoker	17 (12.1)	3 (10.3)	14 (12.6)	0.739

Values are number of patients (%) or mean ± standard deviation

*EuroSCOREII* European System for Cardiac Operative Risk Evaluation II, *COPD* chronic obstructive pulmonary disease, *CKD* chronic kidney disease, *PAI* psoas muscle area index

Linear regression using psoas muscle area as the response variable indicated that age (*p* < 0.001), BSA (*p* < 0.001), female gender (*p* < 0.001), and hypertension (*p* = 0.013) were significantly correlated with psoas muscle area.

Operative outcomes are shown in Table 2. A bioprosthetic valve was used in all cases. Operation time, cardiopulmonary bypass time, aorta cross-clamp time, effective orifice area, and effective orifice area index were similar in both groups. No patients had a ratio of effective orifice area to BSA < 0.80 cm^2^/m^2^) in postoperative echocardiography. In terms of postoperative complications, the low-PAI group had higher risk of needing prolonged ventilation than did the normal-PAI group (the low-PAI group, 6.9%, vs the normal-PAI group, 0%, *p* = 0.005). Two patients in the low-PAI group who required prolonged ventilation were a 94-year-old male patient with chronic obstructive pulmonary disease and a 71-year-old male with very severe AS who had been on hemodialysis for 24 years. Deep wound infection rates were similar in both groups (the low-PAI group: 1 patient, 3.4%, vs the normal-PAI group: 3 patients, 2.7%, *p* = 0.830).

**Table 2:** Operative outcomes

Variables	Overall (*n* = 140)	Low PAI (*n* = 29)	Normal PAI (*n* = 111)	*p value*
Operation time, min	195.1 ± 38.0	193.6 ± 30.4	195.5 ± 40.8	0.819
CPB time, min	90.7 ± 16.3	92.4 ± 21.6	90.2 ± 14.8	0.518
Aorta cross-clamp time, min	60.8 ± 12.1	60.9 ± 16.1	60.8 ± 11.0	0.975
Effective orifice area, cm^2^	1.81 ± 0.28	1.80 ± 0.25	1.81 ± 0.29	0.792
Effective orifice area index, cm^2^/m^2^	1.22 ± 0.18	1.25 ± 0.18	1.21 ± 0.18	0.372
Complications				
Stroke	2 (1.4)	1 (3.4)	1 (0.9)	0.303
Acute renal failure	2 (1.4)	1 (3.4)	1 (0.9)	0.303
Deep wound infection	4 (2.8)	1 (3.4)	3 (2.7)	0.830
Prolonged ventilation, > 48 h	2 (1.4)	2 (6.9)	0 (0)	0.005
Atrial fibrillation	26 (18.6)	5 (17.2)	21 (18.9)	0.836
Reoperation for bleeding	2 (1.4)	1 (3.4)	1 (0.9)	0.303
Event				
30-day mortality	0 (0)	0 (0)	0 (0)	–
In-hospital mortality	2 (1.4)	2 (6.9)	0 (0)	0.005

Values are numbers of patients (%) or mean ± standard deviation

*CPB* cardiopulmonary bypass, *PAI* psoas muscle area index

Thirty-day mortality rates were zero in both groups. In-hospital mortality totaled 2 cases (1.4%), both in the low-PAI group (*p* = 0.005). In-hospital deaths were caused by pyothorax in an 82-year-old male patient and mediastinitis in a 94-year-old male patient. Mean follow-up was 4.25 years (range, 59 days to 12 years), with a total of 27 patient deaths during the follow-up period. The overall follow-up rate was 90.7%. The follow-up rates were similar in both groups (the low-PAI group, 89.7%, vs the normal-PAI group, 91.0%, *p* = 0.825).

**Fig. 4:**
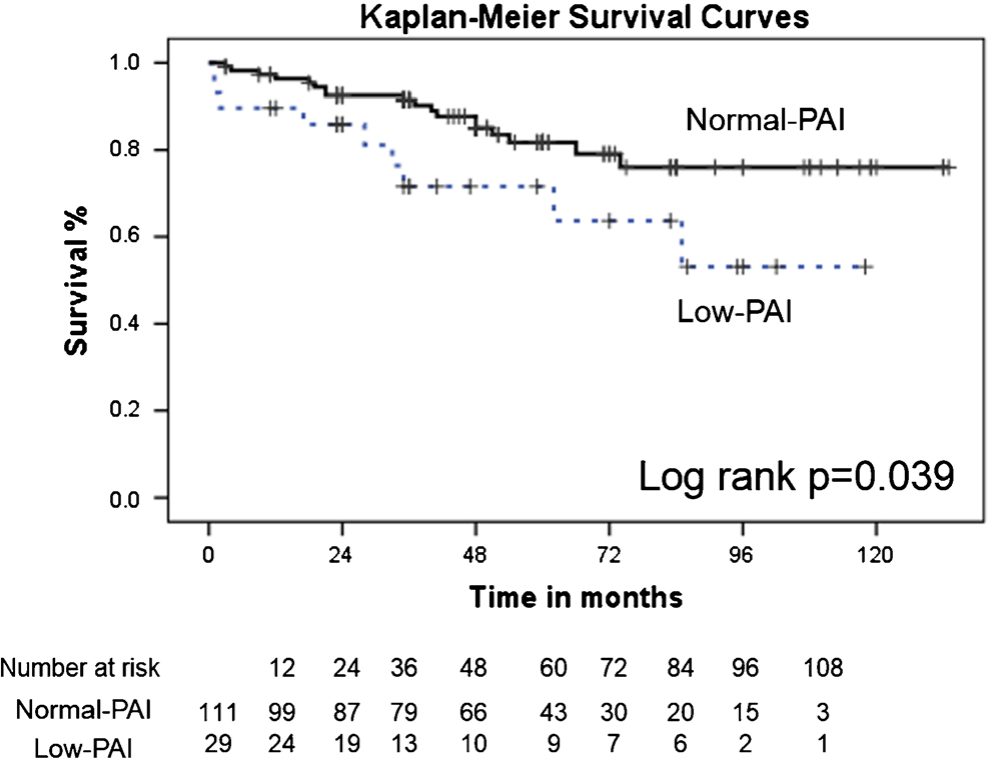
Kaplan–Meier survival analysis by PAI status: normal PAI, solid line; low PAI, dotted line. Numbers at risk are displayed below the figure. Log rank *p* = 0.039. PAI = psoas muscle area index

Kaplan–Meier analysis showed that the low-PAI group had a lower survival rate than the normal-PAI group at 1 year (the low-PAI group, 89.7 ± 5.7%, vs the normal-PAI group, 96.3 ± 1.8%) and at 3 years (the low-PAI group, 71.6 ± 9.3%, vs the normal-PAI group, 91.5 ± 2.7%) and in overall survival (the low-PAI group, 53.0 ± 13.4%, vs the normal-PAI group, 76.0 ± 5.6%, log-rank*p* = 0.039) (Fig. 4). Univariate Cox proportional hazard models showed that low PAI, age, mean pressure gradient, European System for Cardiac Operative Risk Evaluation II grade, chronic kidney disease, and peripheral vascular disease predicted all-cause mortality. Multivariate Cox proportional hazard models showed that low PAI was an independent predictor for all-cause mortality (hazard ratio, 0.235; 95% confidence interval, 0.092–0.602; *p* = 0.003) (Table 3).

**Table 3:** Multivariate analysis of all-cause mortality

Factors	HR	Univariate 95% CI	*p value*	HR	Multivariate 95% CI	*p value*
Age, years	1.110	1.032–1.193	0.005	1.010	0.920–1.108	0.839
Serum albumin, mg/dl	0.238	0.121–0.467	< 0.001	0.235	0.092–0.602	0.003
EuroSCORE II, %	1.330	1.132–1.563	0.001	1.170	0.941–1.455	0.158
CKD	1.330	1.132–1.563	0.001	1.237	0.411–3.723	0.705
Peripheral vascular disease	2.637	0.615–11.31	0.192	1.323	0.245–7.135	0.745
Lowest 20th percentile of PAI^†^	2.273	1.017–5.081	0.045	2.959	1.084–8.079	0.034

*EuroSCOREII* European System for Cardiac Operative Risk Evaluation II; *CKD* chronic kidney disease, serum creatinine > 1.5 mg/dl; *HR* hazard ratio; *CI* confidence interval; *PAI* psoas muscle area index

^†^PAI ≤ 956 mm^2^/m^2^ for males and PAI ≤ 730 mm^2^/m^2^ for females

## Discussion

The main finding of this pilot study was that preoperative low PAI, defined by the gender-specific lowest 20th percentile, was independently associated with worse mid-term mortality after isolated SAVR.

The European Working Group on Sarcopenia in Older People2, which is one of a number of assessment protocols for frailty and sarcopenia, listed the measurable variables as muscle strength (e.g., grip strength, arm strength, leg strength, and chair rise test), muscle quantity (e.g., total body skeletal muscle mass, appendicular skeletal muscle mass, and muscle cross-sectional area of a specific muscle), and physical performance (e.g., gait speed, the short physical performance battery and the Timed Up and Go test). Although CT and magnetic resonance imaging are considered to be gold standards for noninvasive assessment of total body muscle quantity, dual-energy x-ray absorptiometry (DXA) is the preferred alternative for clinical use because of costs and the requirement for highly trained personnel and special software [15]. Dual-energy x-ray absorptiometry (DXA) is a good method to estimate total body muscle mass, but it has disadvantages. First, some DXA instrument brands do not give consistent results, and cross-calibration equations should be used to examine data across systems to avoid erroneous conclusions. Second, DXA measurements can also be influenced by the hydration status of the patient [15]. The hydration status of our patients with cardiac diseases could be changed easily by medication or preoperative status such as edema or heart failure. Third, DXA is unfamiliar to cardiac surgeons and routine preoperative examination. Just using measurements on abdominal CT, we can use psoas muscle area measured by manual tracing on CT as a relatively simple, versatile, objective, and convenient way to assess core muscle size.

Cut-off points for low muscle size vary widely in recent reports, with investigators using parameters such as > 2 standard deviations below the mean among young controls without disease [11], values calculated by receiver operating characteristic curves [10], and the lowest tertile [5, 8, 12], quartile [7, 13, 16, 17], or 20th percentile [18]. That last definition seemed to be the most common among those studies resembling the present study in design, because of lack of data in healthy young adults. We followed that last convention.

Psoas muscle size can be measured in various ways. Two studies showed that psoas muscle volume was a better measure of psoas muscle quantity for sarcopenia than psoas muscle cross-sectional area, for prognosis of postoperative outcomes [16, 17]. Volume studies required specialized software for psoas measurement, so most recent studies have used psoas muscle area rather than volume to judge sarcopenia [5–13]. In the present study, we used psoas muscle area measured by manual tracing on CT as a relatively simple, versatile, objective, and convenient way to assess size.

The level at which to measure the psoas muscle area is also controversial. Kofler and colleagues showed that PAI measured at the level of either the third or fourth lumbar vertebra can be independently related to 30-day mortality and 1-year mortality in patients undergoing TAVR [12]. In their study, PAI was smaller at the third lumbar than at the fourth. They also identified the gender-specific lowest tertile cut-off values of PAI at the L4 level as < 1013 mm^2^/m^2^ in men and < 750 mm^2^/m^2^ in women.

Hawkins and colleagues found that PAI, measured bilaterally at the L4 level, was independently related to 1-year mortality, long-term mortality, and major morbidity in patients undergoing SAVR [7]. Their report identified the gender-specific lowest 25th percentile cut-off values for PAI at the L4 level as < 909 mm^2^/m^2^ in men and < 696 mm^2^/m^2^ in women. These results were similar to ours: ≤ 956 mm^2^/m^2^ for men and ≤ 730 mm^2^/ m^2^ for women, indicating sarcopenia. These facts suggest that PAI may be a widely applicable marker of sarcopenia, not just in a Japanese population.

Since TAVR has become a viable alternative, medical teams need objective measures to choose intervention strategies, and abdominal CT, and hence PAI, is generally readily available whenever TAVR is considered. Now further studies are needed to assess the relationship between PAI and survival after TAVR, but cut-off values will not be available for those choices until adequate data are acquired to calculate the balance of risks between SAVR and TAVR. Currently, one may choose between SAVR and TAVR using both an objective risk score (the Society of Thoracic Surgeons Risk Score) and a possibly less objective frailty assessment (the Canadian Study of Health and Aging Frailty Scale). Thorough assessment of frailty with performance and strength testing requires staff time and patient cooperation, whereas the calculation of PAI takes about 3 min of added operator time and provides an objective measurement related to frailty. While TAVR is still a developing technique and full data on results are not yet available, TAVR seems preferable in higher-risk, frail, elderly patients [19]. Among patients with medium or lower Society of Thoracic Surgeons Risk Scores, however, frailty varies, and moderate or severe frailty may constitute grounds for choosing TAVR over SAVR even in younger cases, whereas SAVR may be viable in the older but less frail cases. If PAI proves sufficiently reliable in relation to TAVR, it may assist the choice of approach. More broadly, however, in the field of postoperative care, PAI may be seen not necessarily through cut-off numbers, but perhaps by reference to distribution charts such as those in this report, to contribute to health-care providers’ assessments of needs for referral for post-dischargerehabilitation/exercise in the community, whether machine-based exercise or other activities.

### Limitations

This study has several limitations. First, it is a retrospective study with unpaired small groups from a single center. Second, its retrospective nature meant that we lacked such data as grip strength and short distance gait speed that would have allowed direct comparison with other measures of sarcopenia or frailty. Third, these data came from only Japanese patients, in the most aging country in the world, and we excluded patients aged below 70 years to take advantage of the prevalence of sarcopenia among older adults after 70 years of age, which is reportedly approximately double that among adults below 70 years of age [20]. This, however, limited the age range for data gathering. Fourth, measurement of the size of one bilateral muscle, such as the psoas muscle, has been used to estimate muscle quantity, but no consensus has been established. Fifth, though greater age at operation (as in the low-PAI group) would be expected to be associated with shorter survival, we were not able to account properly for the difference in patient ages between groups, due to small sample size. Sixth, the findings from this study alone are not enough to direct decisions between SAVR and TAVR; at least one further study will be needed to ascertain the critical PAI values at which SAVR or TAVR may offer lower mortality.

## Conclusions

We analyzed the PAI of 140 elderly patients including 29 low-PAI patients with AS undergoing isolated SAVR. We identified a correlation between gender-specific low PAI and lower overall survival. Thirty-day mortality rates were zero in both groups. Kaplan–Meier analysis showed that the low-PAI group had a lower survival rate than the normal-PAI group at 1 year, at 3 years, and at the mean follow-up period of 4.25 years. With further data, measurement of preoperative PAI may help to choose between SAVR and TAVR for AS patients.

## Data Availability

The data that support the findings of this study are available from the corresponding author, Tomoaki Suzuki, upon reasonable request.

## References

[1] Rockwood K (2005). Frailty and its definition: a worthy challenge. J Am Geriatr Soc.

[2] Brown M, Sinacore DR, Binder EF, Kohrt WM (2000). Physical and performance measures for the identification of mild to moderate frailty. J Gerontol A Biol Sci Med Sci.

[3] Verghese J, Holtzer R, Lipton RB, Wang C. Mobility stress test approach to predicting frailty, disability, and mortality in high‐functioning older adults. J Am Geriatr Soc. 2012;60:1901–5. 10.1111/j.1532-5415.2012.04145.x PMC3470773 23002714

[4] Clegg A, Rogers L, Young J. Diagnostic test accuracy of simple instruments for identifying frailty in community-dwelling older people: a systematic review. Age Ageing. 2015;44:148–52. 10.1093/ageing/afu157 25355618

[5] Lee JS-J, He K, Harbaugh CM, et al. Frailty, core muscle size, and mortality in patients undergoing open abdominal aortic aneurysm repair. J Vasc Surg. 2011;53:912–7. 10.1016/j.jvs.2010.10.111 21215580

[6] Paknikar R, Friedman J, Cron D (2016). Psoas muscle size as a frailty measure for open and transcatheter aortic valve replacement. J Thorac Cardiovasc Surg.

[7] Hawkins RB, Mehaffey JH, Charles EJ (2018). Psoas muscle size predicts risk-adjusted outcomes after surgical aortic valve replacement. Ann Thorac Surg.

[8] Englesbe MJ, Lee JS, He K (2012). Analytic morphomics, core muscle size, and surgical outcomes. Ann Surg.

[9] Mamane S, Mullie L, Piazza N (2016). Psoas muscle area and all-cause mortality after Transcatheter aortic valve replacement: the Montreal-Munich Study. Can J Cardiol.

[10] Heberton GA, Nassif M, Bierhals A (2016). Usefulness of psoas muscle area determined by computed tomography to predict mortality or prolonged length of hospital stay in patients undergoing left ventricular assist device implantation. Am J Cardiol.

[11] Ikeno Y, Koide Y, Abe N (2017). Impact of sarcopenia on the outcomes of elective total arch replacement in the elderly. Eur J Cardiothorac Surg.

[12] Kofler M, Reinstadler SJ, Mayr A, et al. Prognostic implications of psoas muscle area in patients undergoing transcatheter aortic valve implantation. Eur J Cardiothorac Surg. 2019;55:210–216. 10.1093/ejcts/ezy244 30629156

[13] Okamura H, Kimura N, Tanno K, et al. The impact of preoperative sarcopenia, defined based on psoas muscle area, on long-term outcomes of heart valve surgery. J Thorac Cardiovasc Surg. 2019;157:1071–1079.e3. 10.1016/j.jtcvs.2018.06.098 30139644

[14] Beach JM, Mihaljevic T, Svensson LG, et al. Coronary artery disease and outcomes of aortic valve replacement for severe aortic stenosis. J Am Coll Cardiol. 2013;61:837–48. 10.1016/j.jacc.2012.10.049 PMC4262244 23428216

[15] Cruz-Jentoft AJ, Bahat G, Bauer J (2019). Sarcopenia: revised European consensus on definition and diagnosis. Age Ageing.

[16] Amini N, Spolverato G, Gupta R (2015). Impact total psoas volume on short- and long-term outcomes in patients undergoing curative resection for pancreatic adenocarcinoma: a new tool to assess sarcopenia. J Gastrointest Surg.

[17] Valero V, Amini N, Spolverato G (2015). Sarcopenia adversely impacts postoperative complications following resection or transplantation in patients with primary liver tumors. J Gastrointest Surg.

[18] Delmonico MJ, Harris TB, Lee J-S, et al. Alternative definitions of sarcopenia, lower extremity performance, and functional impairment with aging in older men and women. J Am Geriatr Soc. 2007;55:769–74. 10.1111/j.1532-5415.2007.01140.x 17493199

[19] Moss S, Doyle M, Nagaraja V, Peeceeyen S (2020). A systematic review and meta-analysis of the clinical outcomes of TAVI versus SAVR in the octogenarian population. Indian J Thorac Cardiovasc Surg.

[20] Kitamura A, Seino S, Abe T (2021). Sarcopenia: prevalence, associated factors, and the risk of mortality and disability in Japanese older adults. J Cachexia Sarcopenia Muscle.

